# Nomogram combining clinical and radiological characteristics for predicting the malignant probability of solitary pulmonary nodules measuring ≤ 2 cm

**DOI:** 10.3389/fonc.2023.1196778

**Published:** 2023-09-18

**Authors:** Mengchao Xue, Rongyang Li, Kun Wang, Wen Liu, Junjie Liu, Zhenyi Li, Zheng Ma, Huiying Zhang, Hui Tian, Yu Tian

**Affiliations:** Department of Thoracic Surgery, Qilu Hospital of Shandong University, Jinan, China

**Keywords:** solitary pulmonary nodules, radiological characteristics, predictive model, nomogram, diagnosis

## Abstract

**Background:**

At present, how to identify the benign or malignant nature of small (≤ 2 cm) solitary pulmonary nodules (SPN) are an urgent clinical challenge. This retrospective study aimed to develop a clinical prediction model combining clinical and radiological characteristics for assessing the probability of malignancy in SPNs measuring ≤ 2 cm.

**Method:**

In this study, we included patients with SPNs measuring ≤ 2 cm who underwent pulmonary resection with definite pathology at Qilu Hospital of Shandong University from January 2020 to December 2021. Clinical features, preoperative biomarker results, and computed tomography characteristics were collected. The enrolled patients were randomized at a ratio of 7:3 into a training cohort of 775 and a validation cohort of 331. The training cohort was used to construct the predictive model, while the validation cohort was used to test the model independently. Univariate and multivariate logistic regression analyses were performed to identify independent risk factors. The prediction model and nomogram were established based on the independent risk factors. The receiver operating characteristic (ROC) curve was used to evaluate the identification ability of the model. The calibration power was evaluated using the Hosmer–Lemeshow test and calibration curve. The clinical utility of the nomogram was also assessed by decision curve analysis (DCA).

**Result:**

A total of 1,106 patients were included in this study. Among them, the malignancy rate of SPNs was 85.08% (941/1,106). We finally identified the following six independent risk factors by logistic regression: age, carcinoembryonic antigen, nodule shape, calcification, maximum diameter, and consolidation-to-tumor ratio. The area under the ROC curve (AUC) for the training cohort was 0.764 (95% confidence interval [CI]: 0.714–0.814), and the AUC for the validation cohort was 0.729 (95% CI: 0.647–0.811), indicating that the prediction accuracy of nomogram was relatively good. The calibration curve of the predictive model also demonstrated a good calibration in both cohorts. DCA proved that the clinical prediction model was useful in clinical practice.

**Conclusion:**

We developed and validated a predictive model and nomogram for estimating the probability of malignancy in SPNs measuring ≤ 2 cm. With the application of predictive models, thoracic surgeons can make more rational clinical decisions while avoiding overtreatment and wasting medical resources.

## Introduction

With the development and popularity of high-resolution computed tomography (HRCT) as a dominating approach for lung cancer screening, the detection rate of isolated solitary pulmonary nodules (SPNs) has significantly increased in recent years (1–3). Large sample sizes of lung cancer screening trials have shown that the detection rate of SPNs ranges from 8 to 51%, with the vast majority being approximately 20% (4). An SPN is defined as a single, focal, round, hyperdense lung shadow ≤ 3 cm in diameter, surrounded by the lung parenchyma, without pulmonary atelectasis, lymph node enlargement, or pleural effusion (5, 6). Among them, an SPN with a size ≤ 20 mm is defined as small SPN (7). Although SPN size is an independent risk factor for malignancy (8–10), approximately 67.5–78% of small SPNs are malignant (7). The ability to accurately distinguish the degree of malignancy of an SPN is critical to providing patients with more beneficial and personalized treatment, which is currently a research hotspot and difficult area of clinical work (11).

Screening for lung cancer using low-dose computed tomography is an effective modality that can reduce mortality from lung cancer (12, 13). However, the qualitative diagnosis of SPNs measuring ≤ 2 cm remains a challenge for thoracic surgeons. The fact that the pathological results of small SPNs are usually confirmed by invasive or minimally invasive methods imposes a heavy burden on patients and healthcare systems (14). Therefore, a non-invasive method to identify benign and malignant SPNs is highly beneficial to clinical practice. At present, many factors have been identified to help determine the nature of SPNs before surgery. For example, previous studies have demonstrated the value of combined cytokine and tumor marker assays for the differential diagnosis of benign and malignant SPNs, which can improve the accuracy of early lung cancer diagnosis (15–23). Radiological characteristics, such as consolidation-to-tumor ratio (CTR), nodule diameter, presence of spiculation, and location in the lobe, are also increasingly used in the diagnosis of early lung cancer (9, 24–26). Among them, CTR has been a hotspot in lung cancer imaging research in recent years. Many studies have also confirmed that it can be used as the main reference index for judging the malignancy of early lung cancer and for sub-lobar resection, and it is also an independent correlate of recurrence and prognosis of early lung cancer (27–32).

To date, there have been many predictive models for SPN diagnosis, such as the most classic Mayo model, Brock University model, Peking University People’s (PKUPH) model, VA model, and so on. Most of these models have achieved more than 80% diagnostic accuracy. However, each model has its own shortcomings and needs to be further optimized.

The aim of this study was to establish a new predictive model and nomogram to assist in the identification of benign and malignant SPNs measuring ≤ 2 cm based on clinical characteristics, imaging features, and hematological biomarkers, which can help thoracic surgeons make more rational clinical decisions.

## Patients and methods

This single-center study was approved by the Institutional Review Board of Qilu Hospital of Shandong University (registration number: KYLL-202008-023-1). Owing to the retrospective nature of the study, the need for written informed consent was waived. All methods were performed in accordance with the Declaration of Helsinki.

### Patient selection

This was a retrospective study of patients with small SPNs who underwent minimally invasive pulmonary resection with definite pathological results from January 2020 to December 2021 at Qilu Hospital of Shandong University. The inclusion criteria were as follows: (1) patients with a single intra-pulmonary nodule suggested by chest computed tomography (CT) within 1 month before surgery, (2) SPN with a maximum diameter ≤ 2 cm, (3) absence of pulmonary atelectasis and active inflammatory imaging in the lung, and (4) clear pathological findings obtained by surgical resection. The exclusion criteria were as follows: (1) patients aged< 18 years, (2) patients undergoing thoracotomy, (3) incomplete perioperative data, and (4) patients with a history of other malignant disease within 5 years.

All enrolled patients were randomly assigned to the training cohort and validation cohort at a ratio of 7:3 using a random split sample method. The training cohort was used to develop the prediction nomogram, while the validation cohort was used to test the performance of the nomogram.

### Data collection and variable definitions

Eligible patients’ data were collected from the database of Qilu Hospital of Shandong University as follows: (1) demographic data: age, sex, body mass index, smoking history, and preoperative comorbidities such as hypertension, diabetes, and chronic obstructive pulmonary disease (COPD); (2) pre-operative assessment data: percentage of the predicted forced expiratory volume in 1 second, percentage of the predicted value of maximal voluntary ventilation, and American Society of Anesthesiologists score; (3) laboratory blood test indicators: lactate dehydrogenase, serum amyloid, serum 5′-nucleotidase, blood sugar, serum complement C1q, blood type, albumin, lymphocytes, neutrophils, eosinophils, basophils, monocytes, erythrocytes, hemoglobin, platelets, derivative prognostic nutritional index (PNI), neutrophil-lymphocyte ratio (NLR), platelet-lymphocyte ratio (PLR), monocyte-lymphocyte ratio (MLR), derived neutrophil-to-lymphocyte ratio (dNLR), neutrophil to lymphocyte and platelet ratio (NLPR), systemic inflammatory response syndrome, the aggregate index of systemic inflammation (AISI), systemic inflammation index (SII), and pan-immune-inflammation value (PIV); (4) lung cancer tumor markers: pro-gastrin-releasing peptide, squamous cell carcinoma, cytokeratin 19-fragments, carcinoembryonic antigen (CEA), carcinoma antigen 125, and neuron-specific enolase; (5) imaging characteristics on CT: nodule location (centrality or peripherality), nodule shape (regular or not), spiculation (sunburst appearance), cavitation signs, calcification, vascular penetration sign, pleural adhesions, bronchus sign, lobulation, lymph node enlargement sign, pleural effusion sign, maximum tumor diameter, and CTR; and (6) postoperative pathological results.

The period of blood collection from patients in this study was standardized, and all patients had their blood obtained in a fasting and tranquil condition on the morning of the second day of hospitalization. All patients’ blood test results were acquired within one week before surgery.

All chest CT tests, including the whole chest scan, were performed in the supine position. Single scans were taken while holding one’s breath and breathing deeply. The measures were taken by two radiologists with more than five years of experience in chest radiology. Two radiologists independently measured each imaging feature, and any discrepancies were reevaluated by a third radiologist with more than 20 years of experience in chest radiography. Consensus was used to resolve conflicts. The centrality of location was defined as an SPN measuring ≤ 2 cm being located within the inner two-thirds of the lung parenchyma on axial CT images, while peripherality was defined as a nodule located within the outer third. Spiculation was defined as strands that spread from the nodal margins into the lung parenchyma but did not contact the pleural surface. Cavitation signs were defined as gas-filled spaces considered transparent or low-attenuation regions. Calcification was defined as having one of the following patterns on CT imaging: stratification, central nodule, diffusion, or popcorn pattern. The vascular penetration sign was defined as the presence of a vessel crossing the node observed on CT images. Linear attenuation toward the pleura or the primary or secondary fissure from the SPN is known as a pleural adhesion. Direct bronchial involvement of nodules is known as the CT bronchial sign. Lobulation was defined as a wavy or scalloped portion of the lesion surface and strands extending from the nodal margins into the lung parenchyma. The lymph node enlargement sign was an enlargement of the mediastinal lymph nodes that can be observed on the CT image. Lymph node enlargement is defined as a short axis of lymph nodes > 1 cm on CT images. The pleural effusion sign was defined as a blunting of the angle of the rib diaphragm visible on CT images. CTR was the ratio of the diameter of the solid component of the pulmonary nodule to the maximum diameter of the nodule. PNI, NLR, dNLR, MLR, NLPR, SIRI, AISI, SII, and PIV were calculated using the following formulas:


PNI = serum albumin (g/L) + 5 × total lymphocytes (× 109/L)

NLR = neutrophils/lymphocyte

PLR = platelets/lymphocytes

dNLR = neutrophils/(leukocytes neutrophils)

MLR = monocytes/lymphocytes

NLPR = neutrophils/(lymphocytes × platelets)

SIRI = (neutrophils × monocytes)/lymphocytes

AISI = (neutrophils × monocytes × platelets)/lymphocytes

SII = (neutrophils × platelets)/lymphocytes

PIV = (neutrophils × platelets × monocytes)/lymphocytes

### Establishment of the predictive model

Data from the training cohort was analyzed using univariate analysis to assess all factors affecting the probability of SPN malignancy. Then, to find independent predictors, multivariate logistic regression was performed. All factors with P values less than 0.05 in the univariate analysis were included in further multivariate logistic regression analysis. R statistical software (Windows version 4.2.1, http://www.r-project.org/) was used to create the prediction model and nomogram introducing meaningful independent risk factors in the multivariate analysis. A score for each variable was calculated using the regression model, and the predicted probability of malignancy could be derived by summing the scores of the individual variables.

### Predictive model and nomogram performance

The performance of the predictive nomogram was assessed by discriminatory power, calibration, and clinical utility. Discriminative power is the ability of a model to correctly distinguish between events and non-events. We used receiver operating characteristic (ROC) curves to assess the identification efficiency of the predictive nomogram (33). Calibration measures how well the predicted probabilities agree with the actual results. The Hosmer–Lemeshow test was used to assess the calibration capability, with a P value greater than 0.05 indicating satisfactory calibration (34). Then, a nomogram calibration curve was formed to further evaluate the calibration. Internal validation was performed by using a bootstrapping method that was repeated 1,000 times (35). Decision curve analysis (DCA) was used to assess the clinical utility of the predictive nomogram based on the net benefits of different threshold probabilities (36). Based on the ROC curve analysis of the training cohort, the optimal cutoff value was determined when the Youden index (sensitivity + specificity - 1) reached its maximum value.

### Statistical analysis

All statistical analyses were performed using SPSS 26.0 (SPSS Inc., Chicago, Illinois, USA) and R statistical software (Windows version 4.2.1, http://www.r-project.org/). Normally distributed continuous variables were expressed as the mean ± standard deviation and compared using Student’s t-test. For non-normally distributed continuous variables, the data were expressed as median (interquartile range) and compared by the Mann–Whitney U test between the two groups. Categorical variables were compared using Pearson’s chi-square test or Fisher’s exact test. Bilateral P values of< 0.05 were considered statistically significant.

## Results

### Patient characteristics

**Figure 1:**
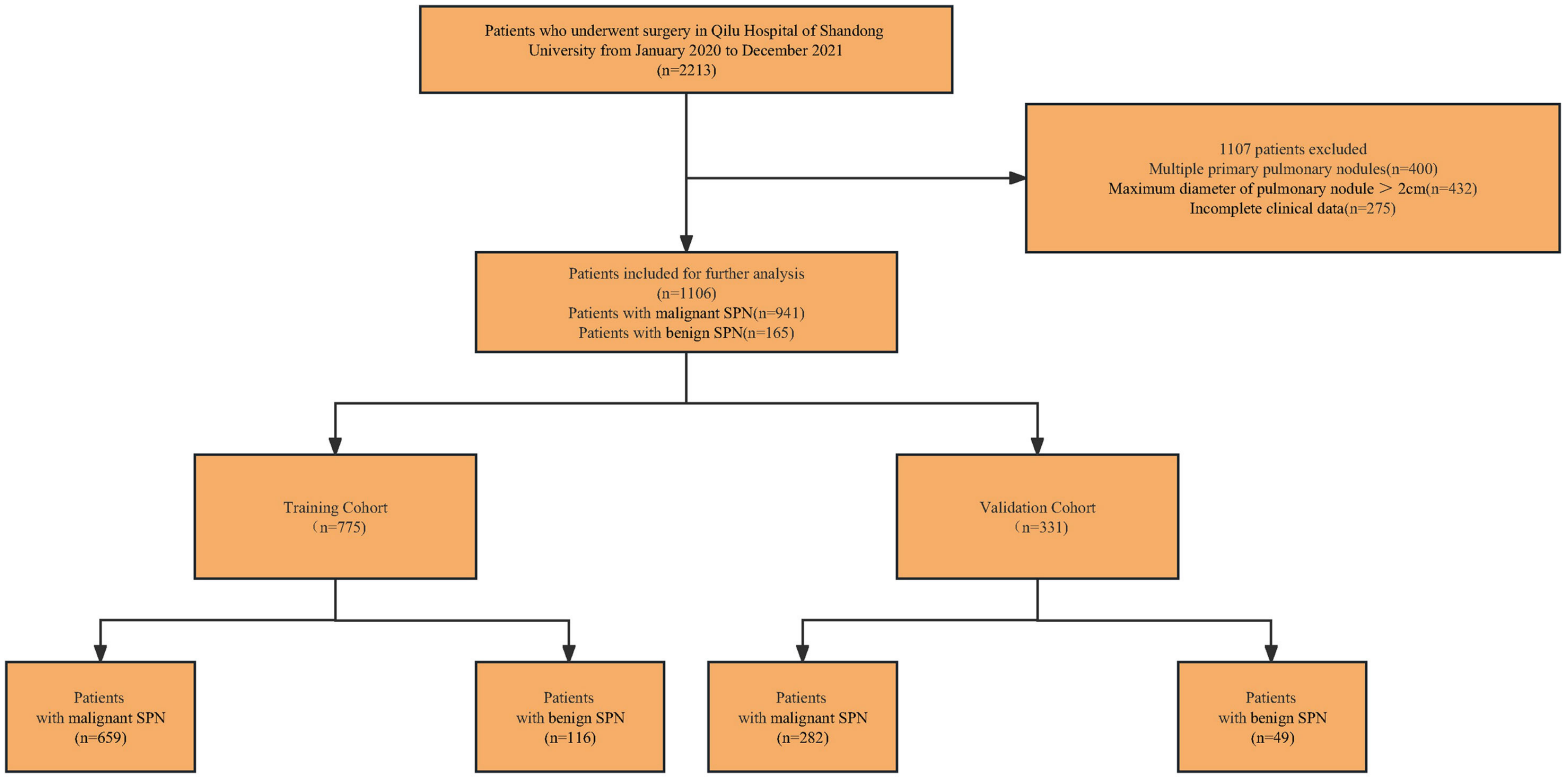
Flow diagram of patient selection through the study. SPN, solitary pulmonary nodules.

The procedure for identifying and selecting eligible patients is shown in **Figure 1**. Our study initially included 2213 initial patients who underwent surgery from January 2020 to December 2021 at our hospital. All initial patients were consecutive and were not selected. A total of 1,106 eligible patients were included in our study after a cascade of screening. Among them, the malignancy rate of SPNs measuring ≤ 2 cm was 85.08% (941/1106). Enrolled patients were then randomly assigned to either the training cohort (n = 775) or validation cohort (n = 331) at a ratio of 7:3, and there were no significant differences in all variables between the two cohorts (**Table 1**). Patients were divided into malignant and benign groups according to the malignancy or non-malignancy of SPNs. The characteristics of the two groups in the training and validation cohorts are shown in **Table 2**.

**Table 1:** Patients’ characteristics of the training cohort and validation cohort.

Characteristics	All cohort (N=1106)	Validation cohort (N=331)	Training cohort (N=775)	P^†^
Malignancy, n (%)				0.944
No	165 (14.9)	49 (14.8)	116 (15.0)	
Yes	941 (85.1)	282 (85.2)	659 (85.0)	
Gender, n (%)				0.455
Female	663 (59.9)	204 (61.6)	459 (59.2)	
Male	443 (40.1)	127 (38.4)	316 (40.8)	
Hypertension, n (%)				0.231
No	798 (72.2)	247 (74.6)	551 (71.1)	
Yes	308 (27.8)	84 (25.4)	224 (28.9)	
Diabetes, n (%)				0.459
No	970 (87.7)	294 (88.8)	676 (87.2)	
Yes	136 (12.3)	37 (11.2)	99 (12.8)	
COPD, n (%)				0.491
No	1096 (99.1)	329 (99.4)	767 (99.0)	
Yes	10 (0.9)	2 (0.6)	8 (1.0)	
Smoking history, n (%)				0.618
Non-smoker	856 (77.4)	253 (76.4)	603 (77.8)	
Smoker	250 (22.6)	78 (23.6)	172 (22.2)	
Blood type, n (%)				0.504
A	336 (30.4)	93 (28.1)	243 (31.4)	
B	373 (33.7)	111 (33.5)	262 (33.8)	
AB	127 (11.5)	37 (11.2)	90 (11.6)	
O	270 (24.4)	90 (27.2)	180 (23.2)	
ASA, n (%)				0.545
1	123 (11.1)	37 (11.2)	86 (11.1)	
2	954 (86.3)	288 (87.0)	666 (85.9)	
3	29 (2.6)	6 (1.8)	23 (3.0)	
Location, n (%)				0.729
Centrality	102 (9.2)	29 (8.8)	73 (9.4)	
Peripherality	1004 (90.8)	302 (91.2)	702 (90.6)	
Shape, n (%)				0.957
Regularity	536 (48.5)	160 (48.3)	376 (48.5)	
Irregularity	570 (51.5)	171 (51.7)	399 (51.5)	
Spiculation, n (%)				0.141
No	495 (44.8)	137 (41.4)	358 (46.2)	
Yes	611 (55.2)	194 (58.6)	417 (53.8)	
Cavitation sign, n (%)				0.637
No	931 (84.2)	276 (83.4)	655 (84.5)	
Yes	175 (15.8)	55 (16.6)	120 (15.5)	
Calcification, n (%)				0.485
No	1092 (98.7)	328 (99.1)	764 (98.6)	
Yes	14 (1.3)	3 (0.9)	11 (1.4)	
Vascular penetration sign, n (%)				0.282
No	387 (35.0)	108 (32.6)	279 (36.0)	
Yes	719 (65.0)	223 (67.4)	496 (64.0)	
Pleural adhesions, n (%)				0.773
No	562 (50.8)	166 (50.2)	396 (51.1)	
Yes	544 (49.2)	165 (49.8)	379 (48.9)	
Bronchus sign, n (%)				0.515
No	869 (78.6)	256 (77.3)	613 (79.1)	
Yes	237 (21.4)	75 (22.7)	162 (20.9)	
Lobulation, n (%)				0.682
No	715 (64.6)	211 (63.7)	504 (65.0)	
Yes	391 (35.4)	120 (36.3)	271 (35.0)	
Lymph node enlargement sign, n (%)				0.377
No	950 (85.9)	289 (87.3)	661 (85.3)	
Yes	156 (14.1)	42 (12.7)	114 (14.7)	
Pleural effusion sign, n (%)				0.937
No	1099 (99.4)	329 (99.4)	770 (99.4)	
Yes	7 (0.6)	2 (0.6)	5 (0.6)	
Albumin (g/L), median (IQR)	59.90 (57.70, 62.10)	59.70 (57.60, 62.30)	60.00 (57.80, 62.00)	0.766
Lymphocyte (×109/L), median (IQR)	1.80 (1.47, 2.21)	1.80 (1.49, 2.22)	1.81 (1.46, 2.20)	0.915
PNI (%), median (IQR)	69.18 (66.25, 71.85)	69.30 (66.47, 71.90)	69.15 (66.22, 71.80)	0.838
Neutrophil (×109/L), median (IQR)	2.99 (2.45, 3.75)	3.04 (2.46, 3.84)	2.97 (2.44, 3.70)	0.201
Eosinophil (×109/L), median (IQR)	0.10 (0.06, 0.17)	0.10 (0.06, 0.16)	0.10 (0.07, 0.17)	0.11
Basophil (×109/L), median (IQR)	0.03 (0.02, 0.04)	0.03 (0.02, 0.04)	0.03 (0.02, 0.04)	0.201
Monocyte (×109/L), median (IQR)	0.41 (0.34, 0.50)	0.41 (0.33, 0.49)	0.41 (0.34, 0.51)	0.205
Erythrocyte (×1012/L), median (IQR)	4.49 (4.19, 4.82)	4.43 (4.17, 4.80)	4.50 (4.21, 4.82)	0.189
Hemoglobin (g/L), median (IQR)	137.00 (128.00, 148.00)	135.00 (128.00, 147.00)	137.00 (128.00, 148.00)	0.444
Platelet (×109/L), median (IQR)	235.00 (200.00, 270.00)	237.00 (197.00, 271.00)	234.00 (201.00, 270.00)	0.784
NLR (%), median (IQR)	1.67 (1.29, 2.12)	1.71 (1.29, 2.22)	1.65 (1.30, 2.09)	0.336
PLR (%), median (IQR)	130.18 (104.77, 158.70)	130.22 (105.49, 157.06)	130.08 (103.98, 159.74)	0.786
MLR (%), median (IQR)	0.22 (0.18, 0.28)	0.22 (0.18, 0.27)	0.22 (0.18, 0.29)	0.24
dNLR (%), median (IQR)	1.26 (1.00, 1.56)	1.29 (1.01, 1.61)	1.25 (1.00, 1.54)	0.096
NLPR (%), median (IQR)	0.01 (0.01, 0.01)	0.01 (0.01, 0.01)	0.01 (0.01, 0.01)	0.32
SIRI (%), median (IQR)	0.66 (0.48, 0.96)	0.66 (0.46, 0.98)	0.66 (0.48, 0.95)	0.855
AISI (%), median (IQR)	153.06 (104.71, 232.70)	152.09 (104.60, 235.80)	154.55 (104.76, 231.97)	0.955
SII (%), median (IQR)	382.56 (289.63, 515.89)	387.84 (289.48, 530.93)	380.30 (291.01, 508.51)	0.381
PIV (%), median (IQR)	153.06 (104.71, 232.70)	152.09 (104.60, 235.80)	154.55 (104.76, 231.97)	0.955
Blood sugar(mmol/L), median (IQR)	5.12 (4.73, 5.66)	5.12 (4.74, 5.64)	5.11 (4.72, 5.68)	0.917
Complement C1q(mg/L), median (IQR)	171.60 (151.67, 191.28)	171.40 (153.85, 191.95)	171.80 (151.15, 191.20)	0.514
LDH (U/L), median (IQR)	192.00 (172.00, 215.00)	195.00 (169.00, 218.50)	191.00 (173.00, 214.00)	0.835
SA (mg/dL), median (IQR)	53.90 (49.30, 58.20)	54.00 (48.80, 58.30)	53.80 (49.60, 57.90)	0.821
5’-NT (U/L), median (IQR)	4.00 (3.00, 5.00)	4.00 (3.00, 5.00)	4.00 (3.00, 5.00)	0.558
Pro-GRP (pg/mL), median (IQR)	41.96 (34.08, 45.92)	41.96 (34.18, 43.75)	41.96 (34.01, 46.34)	0.34
SCC (ng/mL), median (IQR)	1.08 (0.80, 1.97)	1.05 (0.72, 1.94)	1.09 (0.80, 1.97)	0.364
Cyfra21-1 (ng/mL), median (IQR)	2.32 (1.69, 2.56)	2.32 (1.65, 2.58)	2.32 (1.71, 2.55)	0.747
CEA (ng/mL), median (IQR)	2.32 (1.51, 2.64)	2.32 (1.42, 2.70)	2.32 (1.52, 2.62)	0.617
CA125 (U/mL), median (IQR)	10.72 (7.61, 11.38)	10.72 (7.64, 11.70)	10.72 (7.54, 11.30)	0.512
NSE (ng/mL), median (IQR)	19.45 (15.80, 20.50)	19.45 (15.80, 20.40)	19.45 (15.80, 20.60)	0.604
Age (years), median (IQR)	57.00 (50.00, 65.00)	58.00 (51.00, 65.50)	57.00 (50.00, 64.00)	0.531
BMI (kg/m2), median (IQR)	24.77 (22.77, 26.90)	24.72 (22.44, 26.71)	24.80 (22.86, 26.95)	0.217
FEV1% predicted (%), median (IQR)	105.32 (94.89, 115.71)	105.50 (95.98, 115.93)	105.26 (94.43, 115.63)	0.417
MVV% predicted (%), median (IQR)	103.90 (91.37, 116.17)	104.00 (91.13, 116.40)	103.89 (91.40, 116.12)	0.661
Maximum diameter (cm), median (IQR)	1.20 (0.80, 1.50)	1.20 (0.90, 1.50)	1.20 (0.80, 1.50)	0.688
CTR (%), median (IQR)	0.00 (0.00, 0.71)	0.22 (0.00, 0.73)	0.00 (0.00, 0.71)	0.16

COPD, chronic obstructive pulmonary diseases; ASA, American Society of Anesthesiologists; PNI, prognostic nutritional index; NLR, neutrophil-lymphocyte ratio; PLR, platelet-lymphocyte ratio; MLR, monocyte-lymphocyte ratio; dNLR, derived neutrophil-to-lymphocyte ratio; NLPR, neutrophil to lymphocyte and platelet ratio; SIRI, systemic inflammatory response syndrome; AISI, aggregate index of systemic inflammation; SII, systemic inflammation index; PIV, pan-immune-inflammation value; LDH, lactate dehydrogenase; SA, serum amyloid; 5’-NT, 5’-nucleotidase; Pro-GRP, pro-gastrin-releasing peptide; SCC, squamous cell carcinoma; Cyfra21-1, cytokeratin 19-fragments; CEA, carcinoembryonic antigen; CA125, carcinoma antigen 125; NSE, neuron-specific enolase; BMI, body mass index; FEV1, forced expiratory volume in one second; MVV, maximal voluntary ventilation; CTR, consolidation-to-tumor ratio; ^†^ P-value for the comparison between training cohort and validation cohort.

**Table 2:** Clinical characteristics of patients with benign and malignant SPNs measuring ≤ 2cm in the training cohort and validation cohort.

Characteristics	Training Cohort(n=775)			Validation cohort(n=331)		
	Benign(N=122)	Malignancy(N=653)	p	Benign (N=43)	Malignancy(N=288)	p
Gender, n (%)			0.393			0.4
Female	68 (55.7)	391 (59.9)		24 (55.8)	180 (62.5)	
Male	54 (44.3)	262 (40.1)		19 (44.2)	108 (37.5)	
Hypertension, n (%)			0.055			0.911
No	96 (78.7)	458 (70.1)		32 (74.4)	212 (73.6)	
Yes	26 (21.3)	195 (29.9)		11 (25.6)	76 (26.4)	
Diabetes, n (%)			0.505			0.057
No	110 (90.2)	575 (88.1)		33 (76.7)	252 (87.5)	
Yes	12 (9.8)	78 (11.9)		10 (23.3)	36 (12.5)	
COPD, n (%)			0.47			0.584
No	120 (98.4)	647 (99.1)		43 (100.0)	286 (99.3)	
Yes	2 (1.6)	6 (0.9)		0 (0.0)	2 (0.7)	
Smoking history, n (%)			0.34			0.819
Non-smoker	97 (79.5)	493 (75.5)		34 (79.1)	232 (80.6)	
Smoker	25 (20.5)	160 (24.5)		9 (20.9)	56 (19.4)	
Blood type, n (%)			0.572			0.742
A	41 (33.6)	198 (30.3)		15 (34.9)	82 (28.5)	
B	44 (36.1)	217 (33.2)		14 (32.6)	98 (34.0)	
AB	13 (10.7)	72 (11.0)		6 (14.0)	36 (12.5)	
O	24 (19.7)	166 (25.4)		8 (18.6)	72 (25.0)	
ASA, n (%)			0.372			0.499
1	15 (12.3)	76 (11.6)		3 (7.0)	29 (10.1)	
2	106 (86.9)	557 (85.3)		38 (88.4)	253 (87.8)	
3	1 (0.8)	20 (3.1)		2 (4.7)	6 (2.1)	
Location, n (%)			0.962			0.697
Centrality	11 (9.0)	58 (8.9)		5 (11.6)	28 (9.7)	
Peripherality	111 (91.0)	595 (91.1)		38 (88.4)	260 (90.3)	
Shape, n (%)			0.021			0.486
Regularity	72 (59.0)	311 (47.6)		22 (51.2)	131 (45.5)	
Irregularity	50 (41.0)	342 (52.4)		21 (48.8)	157 (54.5)	
Spiculation, n (%)			0.069			0.093
No	63 (51.6)	279 (42.7)		25 (58.1)	128 (44.4)	
Yes	59 (48.4)	374 (57.3)		18 (41.9)	160 (55.6)	
Cavitation sign, n (%)			0.713			0.734
No	104 (85.2)	548 (83.9)		37 (86.0)	242 (84.0)	
Yes	18 (14.8)	105 (16.1)		6 (14.0)	46 (16.0)	
Calcification, n (%)			<0.001			0.135
No	116 (95.1)	651 (99.7)		41 (95.3)	284 (98.6)	
Yes	6 (4.9)	2 (0.3)		2 (4.7)	4 (1.4)	
Vascular penetration sign, n (%)			0.085			0.645
No	49 (40.2)	210 (32.2)		18 (41.9)	110 (38.2)	
Yes	73 (59.8)	443 (67.8)		25 (58.1)	178 (61.8)	
Pleural adhesions, n (%)			0.47			0.426
No	66 (54.1)	330 (50.5)		24 (55.8)	142 (49.3)	
Yes	56 (45.9)	323 (49.5)		19 (44.2)	146 (50.7)	
Bronchus sign, n (%)			0.107			0.156
No	103 (84.4)	509 (77.9)		37 (86.0)	220 (76.4)	
Yes	19 (15.6)	144 (22.1)		6 (14.0)	68 (23.6)	
Lobulation, n (%)			0.953			0.645
No	80 (65.6)	430 (65.8)		28 (65.1)	177 (61.5)	
Yes	42 (34.4)	223 (34.2)		15 (34.9)	111 (38.5)	
Lymph node enlargement sign, n (%)			0.52			0.401
No	108 (88.5)	564 (86.4)		38 (88.4)	240 (83.3)	
Yes	14 (11.5)	89 (13.6)		5 (11.6)	48 (16.7)	
Pleural effusion sign, n (%)			0.95			0.699
No	121 (99.2)	648 (99.2)		43 (100.0)	287 (99.7)	
Yes	1 (0.8)	5 (0.8)		0 (0.0)	1 (0.3)	
Albumin (g/L), median (IQR)	59.75 (57.60, 62.38)	59.90 (57.70, 62.00)	0.89	60.20 (57.40, 62.20)	60.00 (57.88, 62.10)	0.981
Lymphocyte (×109/L), median (IQR)	1.76 (1.48, 2.27)	1.80 (1.48, 2.19)	0.81	1.85 (1.42, 2.26)	1.81 (1.46, 2.21)	0.777
PNI (%), median (IQR)	69.28 (66.31, 72.55)	69.10 (66.25, 71.60)	0.707	69.45 (66.25, 71.60)	69.30 (66.27, 71.86)	0.791
Neutrophil (×109/L), median (IQR)	2.98 (2.34, 3.61)	2.97 (2.45, 3.79)	0.52	2.97 (2.49, 3.66)	3.04 (2.46, 3.73)	0.757
Eosinophil (×109/L), median (IQR)	0.12 (0.07, 0.16)	0.10 (0.06, 0.17)	0.413	0.09 (0.06, 0.14)	0.10 (0.06, 0.19)	0.212
Basophil (×109/L), median (IQR)	0.03 (0.02, 0.04)	0.03 (0.02, 0.04)	0.994	0.03 (0.02, 0.04)	0.03 (0.02, 0.04)	0.09
Monocyte (×109/L), median (IQR)	0.39 (0.33, 0.51)	0.41 (0.34, 0.50)	0.993	0.41 (0.36, 0.48)	0.42 (0.34, 0.51)	0.997
Erythrocyte (×1012/L), median (IQR)	4.56 (4.24, 4.91)	4.48 (4.19, 4.82)	0.246	4.48 (4.22, 4.76)	4.49 (4.17, 4.79)	0.965
Hemoglobin (g/L), median (IQR)	138.00 (128.25, 153.00)	137.00 (128.00, 147.00)	0.353	136.00 (127.50, 144.50)	135.50 (127.00, 148.00)	0.845
Platelet (×109/L), median (IQR)	234.00 (190.00, 271.00)	235.00 (202.00, 269.00)	0.355	244.00 (208.00, 275.00)	234.00 (200.00, 270.00)	0.707
NLR (%), median (IQR)	1.62 (1.35, 1.96)	1.65 (1.27, 2.16)	0.419	1.67 (1.35, 2.03)	1.72 (1.32, 2.13)	0.842
PLR (%), median (IQR)	130.60 (102.65, 157.05)	130.00 (104.78, 157.87)	0.544	123.35 (108.66, 159.40)	131.07 (104.67, 163.18)	0.573
MLR (%), median (IQR)	0.22 (0.18, 0.28)	0.22 (0.18, 0.28)	0.959	0.23 (0.20, 0.27)	0.23 (0.18, 0.29)	0.948
dNLR (%), median (IQR)	1.21 (0.99, 1.47)	1.26 (1.00, 1.57)	0.443	1.27 (1.01, 1.56)	1.28 (1.02, 1.55)	0.895
NLPR (%), median (IQR)	0.01 (0.01, 0.01)	0.01 (0.01, 0.01)	0.744	0.01 (0.00, 0.01)	0.01 (0.01, 0.01)	0.803
SIRI (%), median (IQR)	0.63 (0.47, 0.93)	0.65 (0.47, 0.97)	0.631	0.73 (0.49, 0.95)	0.69 (0.50, 0.95)	0.993
AISI (%), median (IQR)	142.28 (99.33, 218.96)	150.35 (101.92, 236.97)	0.469	153.06 (112.59, 223.07)	163.73 (110.22, 231.85)	0.777
SII (%), median (IQR)	372.78 (279.22, 494.62)	383.82 (288.40, 514.40)	0.311	362.11 (289.54, 557.22)	389.61 (303.50, 519.41)	0.608
PIV (%), median (IQR)	142.28 (99.33, 218.96)	150.35 (101.92, 236.97)	0.469	153.06 (112.59, 223.07)	163.73 (110.22, 231.85)	0.777
Blood sugar(mmol/L), median (IQR)	4.96 (4.74, 5.37)	5.17 (4.74, 5.71)	0.007	5.16 (4.69, 6.06)	5.11 (4.70, 5.70)	0.881
Complement C1q(mg/L), median (IQR)	173.40 (154.25, 196.48)	170.90 (151.40, 190.20)	0.199	169.70 (152.80, 192.55)	172.20 (153.32, 191.88)	0.946
LDH (U/L), median (IQR)	188.00 (171.25, 206.75)	192.00 (171.00, 214.00)	0.262	195.89 (176.50, 221.50)	193.00 (172.75, 217.00)	0.646
SA (mg/dL), median (IQR)	53.70 (49.45, 58.13)	54.00 (49.20, 58.30)	0.85	54.03 (49.10, 57.80)	53.15 (49.75, 57.80)	0.769
5’-NT (U/L), median (IQR)	4.00 (4.00, 5.00)	4.00 (3.00, 5.00)	0.284	4.00 (3.00, 5.00)	4.00 (3.00, 5.00)	0.905
Pro-GRP (pg/mL), median (IQR)	41.96 (34.73, 47.73)	41.96 (34.40, 46.10)	0.794	41.96 (34.17, 45.84)	41.96 (33.57, 44.82)	0.971
SCC (ng/mL), median (IQR)	1.08 (0.76, 1.97)	1.10 (0.78, 1.97)	0.966	0.93 (0.71, 1.35)	1.06 (0.80, 1.94)	0.08
Cyfra21-1 (ng/mL), median (IQR)	2.32 (1.63, 2.57)	2.32 (1.70, 2.53)	0.936	2.12 (1.53, 2.53)	2.32 (1.72, 2.62)	0.299
CEA (ng/mL), median (IQR)	2.20 (1.32, 2.38)	2.32 (1.51, 2.63)	0.057	2.25 (1.46, 2.41)	2.32 (1.61, 2.81)	0.171
CA125 (U/mL), median (IQR)	10.35 (7.38, 10.93)	10.72 (7.67, 11.30)	0.321	10.50 (7.48, 11.20)	10.71 (7.69, 11.53)	0.885
NSE (ng/mL), median (IQR)	19.45 (16.65, 21.00)	19.45 (16.00, 20.50)	0.3	17.50 (14.60, 19.45)	19.45 (15.40, 21.02)	0.103
Age (years), median (IQR)	54.50 (47.25, 60.00)	58.00 (50.00, 66.00)	0.001	57.00 (50.00, 61.50)	58.00 (51.75, 65.00)	0.146
BMI (kg/m2), median (IQR)	24.49 (22.51, 26.43)	24.78 (22.67, 26.84)	0.373	24.75 (22.98, 27.27)	24.92 (23.12, 27.24)	0.935
FEV1% predicted (%), median (IQR)	106.16 (97.34, 115.51)	105.33 (94.44, 115.88)	0.466	104.05 (92.55, 115.83)	105.00 (94.21, 114.56)	0.769
MVV% predicted (%), median (IQR)	103.52 (91.66, 118.42)	104.73 (91.39, 116.14)	0.712	109.96 (96.53, 119.33)	101.84 (90.38, 114.64)	0.061
Maximum diameter (cm), median (IQR)	1.00 (0.72, 1.50)	1.20 (0.80, 1.50)	0.027	1.00 (0.80, 1.45)	1.20 (0.90, 1.50)	0.033
CTR (%), median (IQR)	0.66 (0.00, 1.00)	0.00 (0.00, 0.62)	<0.001	0.72 (0.00, 1.00)	0.00 (0.00, 0.69)	0.001

COPD, chronic obstructive pulmonary diseases; ASA, American Society of Anesthesiologists; PNI, prognostic nutritional index; NLR, neutrophil-lymphocyte ratio; PLR, platelet-lymphocyte ratio; MLR, monocyte-lymphocyte ratio; dNLR, derived neutrophil-to-lymphocyte ratio; NLPR, neutrophil to lymphocyte and platelet ratio; SIRI, systemic inflammatory response syndrome; AISI, aggregate index of systemic inflammation; SII, systemic inflammation index; PIV, pan-immune-inflammation value; LDH, lactate dehydrogenase; SA, serum amyloid; 5’-NT, 5’-nucleotidase; Pro-GRP, pro-gastrin-releasing peptide; SCC, squamous cell carcinoma; Cyfra21-1, cytokeratin 19-fragments; CEA, carcinoembryonic antigen; CA125, carcinoma antigen 125; NSE, neuron-specific enolase; BMI, body mass index; FEV1, forced expiratory volume in one second; MVV, maximal voluntary ventilation; CTR, consolidation-to-tumor ratio.

### Identification of risk factors for SPNs measuring ≤ 2 cm

**Figure 2:**
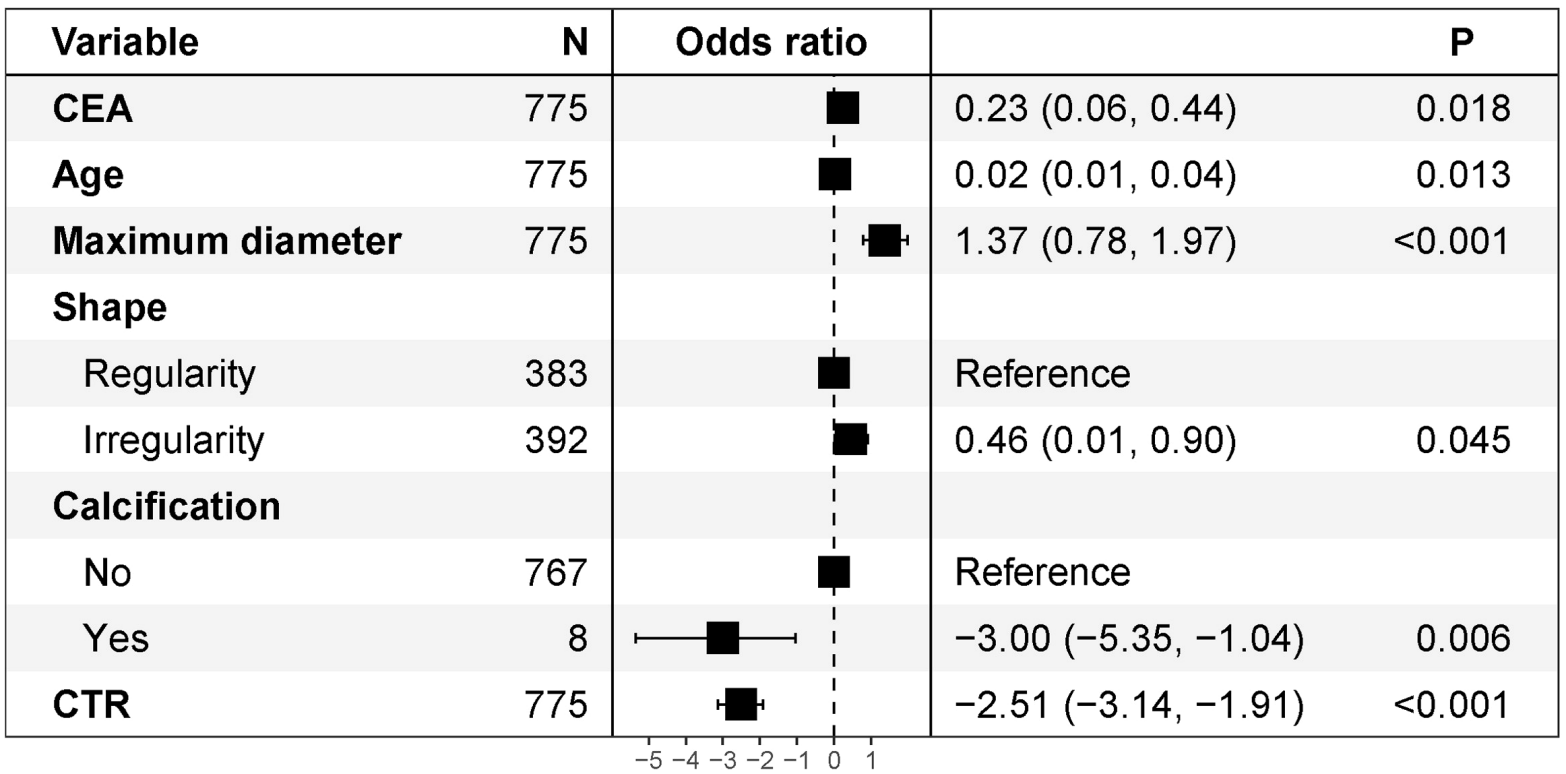
Multivariate logistic regression analysis of forest plots. CEA, carcinoembryonic antigen; CTR, consolidation-to-tumor ratio.

Univariate and multivariate logistic regression analyses were performed in the training cohort to explore independent risk factors for SPN benignity and malignancy (**Table 3**). Univariate analysis showed that age, CEA, shape, calcification, maximum tumor diameter, and CTR were potential risk factors for SPNs measuring ≤ 2 cm (P< 0.05). Further multivariate logistic regression by including variables with a univariate P value< 0.05 showed that CTR (odds ratio [OR] = 0.081; 95% confidence interval [CI]: 0.043–0.147; P< 0.001), calcification (yes vs. no; OR = 0.050; 95% CI: 0.005–0.355; P = 0.006), age (OR = 1.025; 95% CI: 1.005–1.046; P = 0.013), maximum tumor diameter (OR = 3.927; 95% CI: 2.192–7.204; P< 0.001), CEA (OR = 1.265; 95% CI: 1.057–1.556; P = 0.018), and nodule shape (regularity vs. irregularity; OR = 1.577; 95% CI: 1.013–2.470; P = 0.045) were risk factors. The forest plot for the multivariate logistic regression analysis is shown in **Figure 2**.

**Table 3:** Univariate and multivariate logistic regression analysis of the training cohort.

Characteristics	Univariate analysis		Multivariate analysis	
	OR (95%CI)	P value	OR (95%CI)	P value
CTR	0.217 (0.134, 0.347)	<0.001	0.081 (0.043, 0.147)	<0.001
Calcification				
No	Ref.	Ref.	Ref.	Ref.
Yes	0.059 (0.009, 0.261)	0.001	0.050 (0.005, 0.355)	0.006
Age	1.025 (1.007, 1.043)	0.005	1.025 (1.005, 1.046)	0.013
Maximum diameter	1.768 (1.125, 2.809)	0.014	3.927 (2.192, 7.204)	<0.001
CEA	1.234 (1.049, 1.485)	0.019	1.265 (1.057, 1.556)	0.018
Shape				
Regularity	Ref.	Ref.	Ref.	Ref.
Irregularity	1.584 (1.073, 2.354)	0.022	1.577 (1.013, 2.470)	0.045
Hypertension				
No	Ref.	Ref.		
Yes	1.572 (1.002, 2.545)	0.056		
Spiculation				
No	Ref.	Ref.		
Yes	1.431 (0.972, 2.112)	0.07		
Vascular penetration sign				
No	Ref.	Ref.		
Yes	1.416 (0.948, 2.102)	0.086		
Bronchus sign				
No	Ref.	Ref.		
Yes	1.534 (0.928, 2.657)	0.109		
Blood sugar	1.143 (0.965, 1.406)	0.163		
SII	1.001 (1.000, 1.002)	0.181		
IDH	1.004 (0.999, 1.010)	0.194		
ASA				
1	Ref.	Ref.		
2	3.947 (0.730, 73.485)	0.196		
3	1.037 (0.555, 1.826)	0.904		
Blood type				
A	Ref.	Ref.		
B	1.021 (0.639, 1.630)	0.93		
AB	1.147 (0.595, 2.338)	0.693		
O	1.432 (0.837, 2.498)	0.196		
Erythrocyte	0.767 (0.507, 1.155)	0.207		
CA125	1.024 (0.990, 1.068)	0.231		
Platelet	1.002 (0.999, 1.005)	0.248		
BMI	1.037 (0.975, 1.104)	0.249		
Complement C1q	0.997 (0.991, 1.002)	0.255		
AISI	1.001 (1.000, 1.002)	0.302		
PIV	1.001 (1.000, 1.002)	0.302		
FEV1% predicted (%)	0.994 (0.983, 1.006)	0.329		
Smoking history				
Non-smoker	Ref.	Ref.		
Smoker	1.259 (0.795, 2.060)	0.341		
PLR	1.002 (0.998, 1.006)	0.376		
Gender				
Female	Ref.	Ref.		
Male	0.844 (0.572, 1.250)	0.393		
MVV% predicted (%)	0.996 (0.987, 1.005)	0.4		
Pleural adhesions				
NO	Ref.	Ref.		
Yes	1.154 (0.783, 1.704)	0.47		
COPD				
NO	Ref.	Ref.		
Yes	0.556 (0.126, 3.828)	0.476		
dNLR	1.125 (0.825, 1.621)	0.494		
Neutrophil	1.056 (0.909, 1.253)	0.504		
Diabetes				
No	Ref.	Ref.		
Yes	1.243 (0.678, 2.472)	0.505		
Lymph node enlargement sign				
No	Ref.	Ref.		
Yes	1.217 (0.688, 2.304)	0.521		
NLR	1.067 (0.891, 1.339)	0.538		
SIRI	1.073 (0.964, 1.435)	0.538		
Eosinophil	1.544 (0.513, 8.148)	0.539		
Cyfra21_1	1.059 (0.865, 1.326)	0.596		
MLR	1.293 (0.865, 5.907)	0.612		
Monocyte	1.162 (0.922, 3.190)	0.63		
Hemoglobin	0.997 (0.984, 1.010)	0.642		
Basophil	6.129 (0.051, 177932.227)	0.648		
Pro-GRP	0.997 (0.983, 1.012)	0.667		
Cavitation sign				
No	Ref.	Ref.		
Yes	1.107 (0.658, 1.957)	0.713		
PNI	0.994 (0.955, 1.031)	0.738		
5’-NT	0.983 (0.886, 1.114)	0.758		
NSE	0.996 (0.969, 1.027)	0.764		
Albumin	0.994 (0.949, 1.037)	0.795		
SA	1.003 (0.975, 1.033)	0.834		
Lymphocyte	0.968 (0.699, 1.357)	0.847		
Pleural effusion sign				
No	Ref.	Ref.		
Yes	0.934 (0.149, 17.971)	0.95		
Lobulation				
No	Ref.	Ref.		
Yes	0.988 (0.661, 1.494)	0.953		
SCC	0.992 (0.764, 1.338)	0.957		
Location				
No	Ref.	Ref.		
Yes	1.017 (0.492, 1.926)	0.962		
NLPR	1.002 (0.000, 4466896293164250.500)	1		

COPD, chronic obstructive pulmonary diseases; ASA, American Society of Anesthesiologists; PNI, prognostic nutritional index; NLR, neutrophil-lymphocyte ratio; PLR, platelet-lymphocyte ratio; MLR, monocyte-lymphocyte ratio; dNLR, derived neutrophil-to-lymphocyte ratio; NLPR, neutrophil to lymphocyte and platelet ratio; SIRI, systemic inflammatory response syndrome; AISI, aggregate index of systemic inflammation; SII, systemic inflammation index; PIV, pan-immune-inflammation value; LDH, lactate dehydrogenase; SA, serum amyloid; 5’-NT, 5’-nucleotidase; Pro-GRP, pro-gastrin-releasing peptide; SCC, squamous cell carcinoma; Cyfra21-1, cytokeratin 19-fragments; CEA, carcinoembryonic antigen; CA125, carcinoma antigen 125; NSE, neuron-specific enolase; BMI, body mass index; FEV1, forced expiratory volume in one second; MVV, maximal voluntary ventilation; CTR, consolidation-to-tumor ratio.

### Nomogram establishment

**Figure 3:**
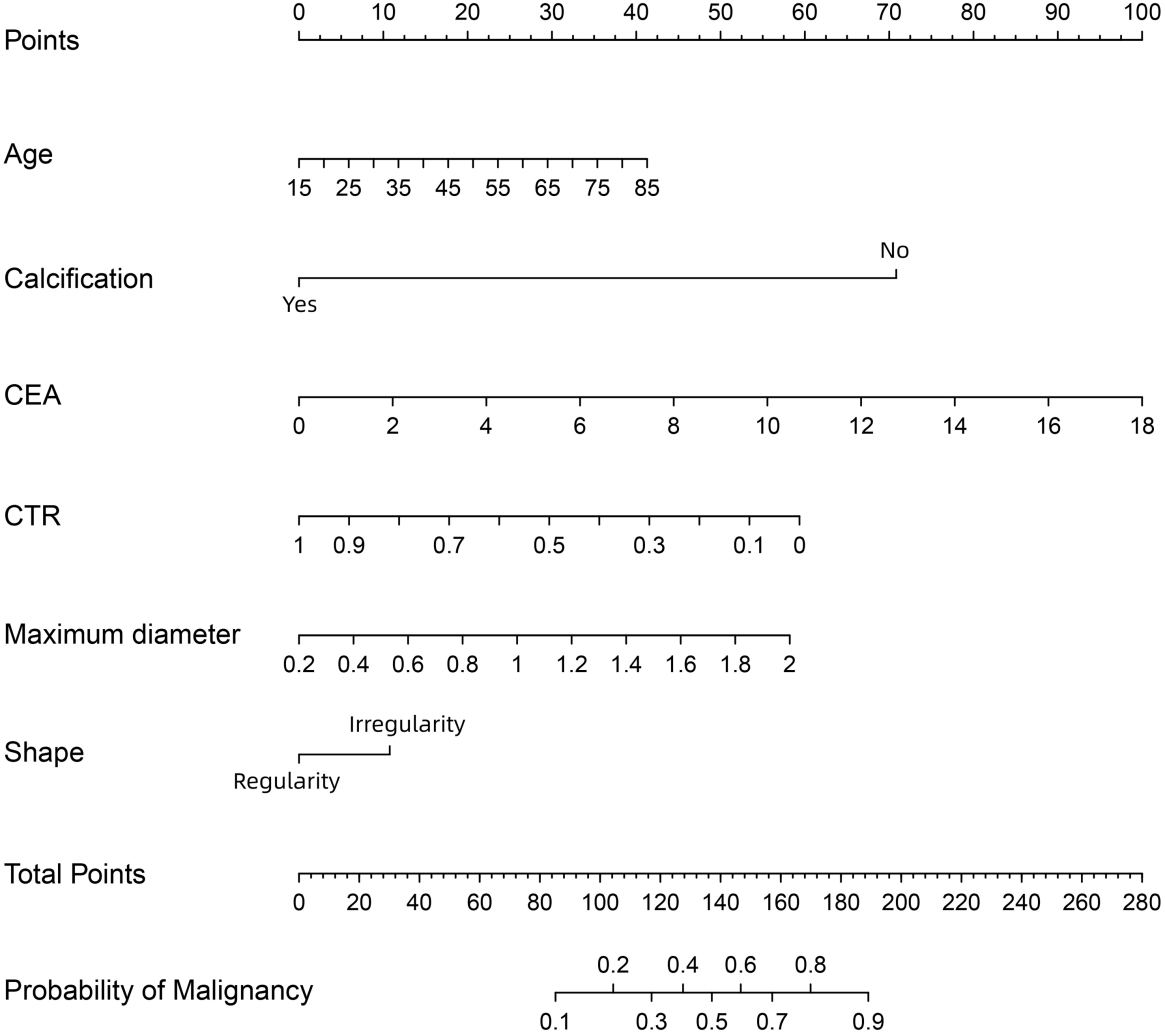
A nomogram for predicting the probability of malignancy in SPN measuring ≤ 2 cm. CEA, carcinoembryonic antigen; CTR, consolidation-to-tumor ratio. There are a total of 9 axes, and axes 2-7 represent the 6 variables in the prediction model. The estimated score for each risk factor can be calculated by plotting a line perpendicular to the highest point axis, and can be further summed to obtain the total score. The total point axis is then used to predict the probability of malignancy for SPN measuring ≤ 2 cm before surgery.

All six independent risk factors for SPNs measuring ≤ 2 cm were included to build the logistic regression model. The predicted probability of malignancy for small SPNs could be calculated by using the following formula: ln (p/1-p) = -2.511 × CTR + 1.368 × maximum diameter - 2.997 × calcification (no = 0; yes = 1) + 0.025 × age + 0.235 × CEA + 0.455 × shape (regularity = 0; irregularity = 1) - 0.941. Based on the above formula, a malignancy probability prediction nomogram for SPNs measuring ≤ 2 cm was drawn using R statistical software (**Figure 3**). As shown in this nomogram, there are a total of nine axes, and axes 2–7 represent the six variables in the prediction model. The estimated score for each risk factor can be calculated by plotting a line perpendicular to the highest point axis and can be further summed to obtain the total score. The total point axis is then used to predict the probability of malignancy for SPNs before surgery, and the appropriate surgical procedure can then be further selected.

### Predictive performance and validation of the nomogram

**Figure 4:**
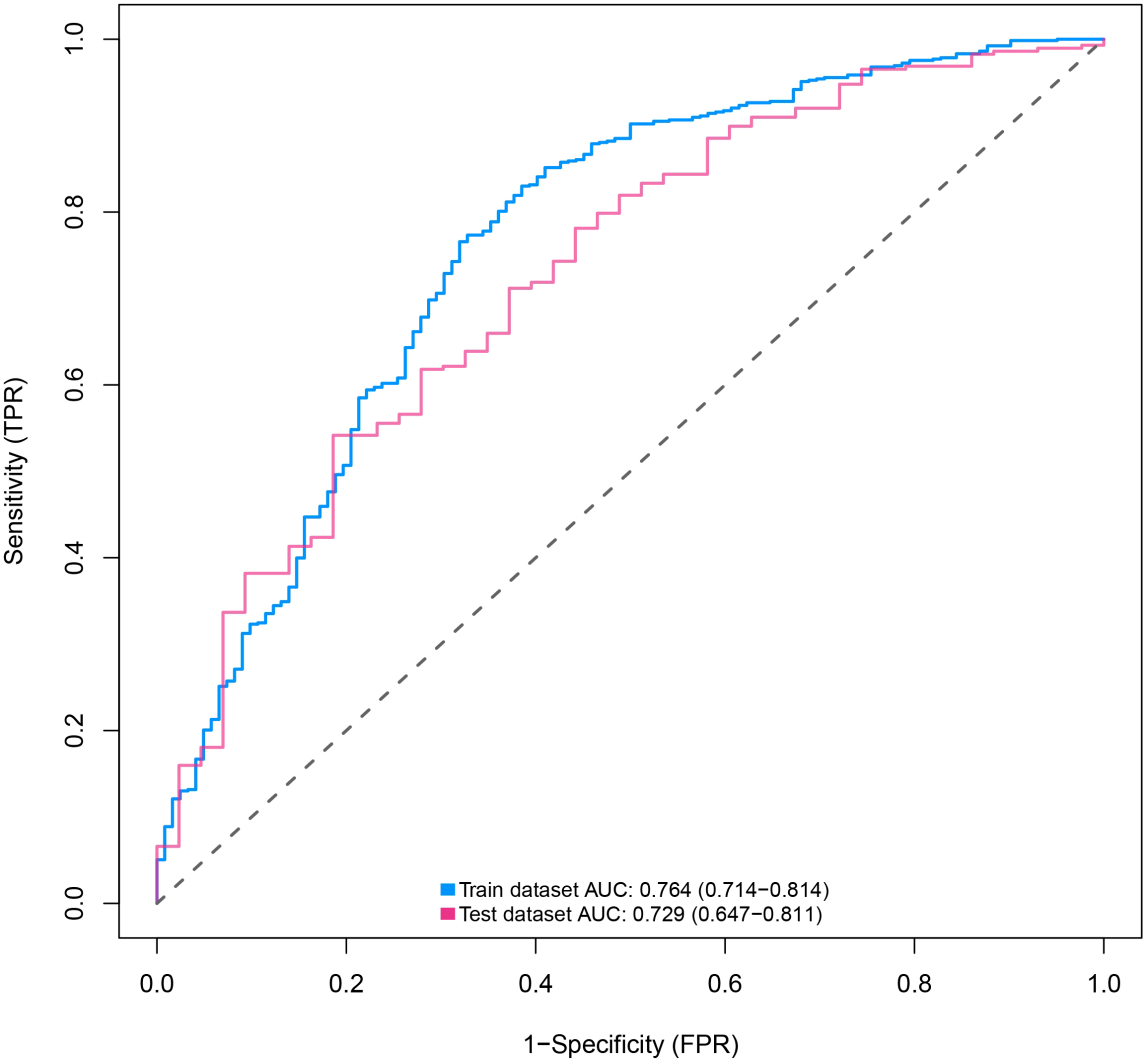
ROC curves of nomograms for predicting the malignancy of SPN within 2 cm in the training and validation cohorts predicting. ROC, receiver operating characteristic; AUC, area under the ROC curve; SPN, solitary pulmonary nodule.

**Figure 5:**
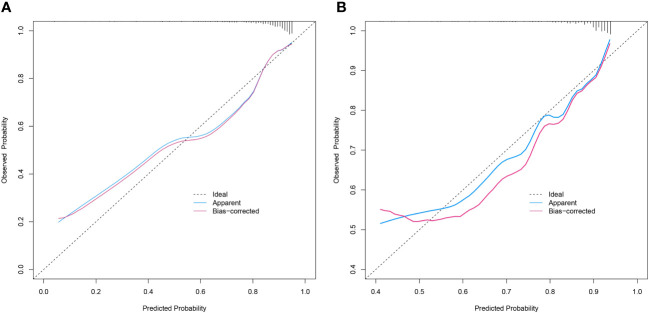
Calibration curves of the prediction nomogram in the training cohort **(A)** and validation cohort **(B)**. The X-axis represents the probability predicted by the nomogram and the Y-axis represents the actual probability of malignancy of SPN within 2 cm. The black dashed line represents the ideal curve, the blue solid line represents the apparent curve (non-corrected), and the red solid line represents the bias-corrected curve by bootstrapping (B = 1000 repetitions). SPN, solitary pulmonary nodule.

The discriminative power of the predictive model and nomogram was assessed by the ROC curve (**Figure 4**). The area under the curve (AUC) for the training cohort was 0.764 (95% CI: 0.714–0.814), and the AUC for the validation cohort was 0.729 (95% CI: 0.647–0.811), indicating a relatively good predictive accuracy of the nomogram. The cut-off value for the ROC curve of the training cohort was 0.819, and the sensitivity and specificity were 0.680 and 0.766, respectively (**Table 4**). Calibration power was evaluated using the Hosmer–Lemeshow test and calibration plots. P values for the Hosmer–Lemeshow test were 0.4348 for the training cohort and 0.3175 for the validation cohort, indicating a negligible difference between the predicted probability and actual observed probability. The calibration plots for the training (**Figure 5A**) and validation (**Figure 5B**) cohorts also demonstrate a good calibration of the predictive nomogram.

**Table 4:** Results of ROC curve for training cohort.

Characteristics	Value
Threshold	0.819
Specificity	0.68
Sensitivity	0.766
Accuracy	0.752
TN	83
TP	500
FN	153
FP	39
NPR	0.352
PPV	0.928
FDR	0.072
FPR	0.32
TPR	0.766
TNR	0.68
FNR	0.234
1-specificity	0.32
1-sensitivity	0.234
1-accuracy	0.248
1-NPV	0.648
1-PPV	0.072
Precision	0.928
Recall	0.766
Youden index	1.446
Closest.topleft	0.157

TP, true positive; FP, false positive; TN, true negative; FN, false negative; TPR, true positive rate; FPR, false positive rate; TNR, true negative rate; FNR, false negative rate; PPV, positive predict value; NPR, negative predict value; FDR, false discovery rate.

### Clinical utility of the predictive nomogram

**Figure 6:**
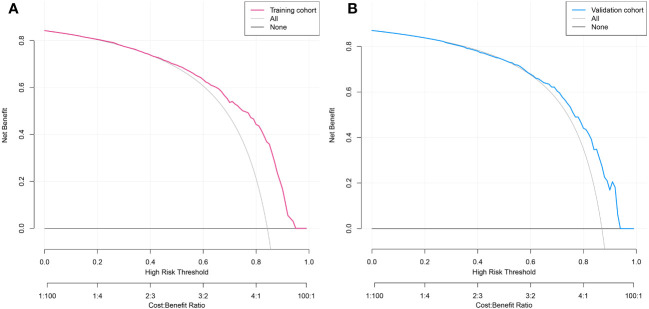
Decision curve analysis of predicted nomogram in the training cohort **(A)** and validation cohort **(B)**. The y-axis measures net gain, with the black line representing the assumption of patients whose SPNs within 2 cm are benign and the gray line representing the assumption of patients whose SPNs measuring ≤2 cm are malignant. The red line in **Figure 6A** represents the training cohort, and the blue line in **Figure 6B** represents the validation cohort.

DCA was used to assess the clinical utility of the predictive nomogram (**Figures 6A, B**). The results show that the nomogram provided greater net benefit and broader threshold probabilities for predicting the risk of malignancy in SPNs measuring ≤ 2 cm in both the training and validation cohorts, indicating that the nomogram is clinically useful.

## Discussion

At present, the most frequent cause of cancer-related death is lung cancer (37–39). Most lung cancers are at an advanced stage when detected and have a poor prognosis. Enhancing the diagnosis rate of early-stage lung cancer to provide proper and rational treatment is crucial to increasing the survival rate (40). Several recent institutional retrospective studies have suggested that survival and recurrence rates may be the same for lobectomy and sub-lobar resection in patients with small lung cancers measuring ≤ 2 cm. Therefore, the management of patients with growing SPNs of 2 cm or smaller is a high priority for clinicians. In this study, we developed a clinical prediction model and designed a nomogram with good predictive performance for assessing the malignancy of small SPNs. This predictive nomogram can be used to estimate the probability of nodal malignancy in patients with SPNs measuring ≤ 2 cm, and thoracic surgeons can make more rational clinical decisions while avoiding overtreatment and wasting medical resources.

In this study, multivariate logistic regression analysis showed that age, CEA, shape, calcification, maximum tumor diameter, and CTR were independent predictors for estimating SPN malignancy. Based on these results, a clinical prediction model for SPNs measuring ≤ 2 cm was developed by incorporating one general clinical indicator (age), four imaging indicators (shape, calcification, maximum tumor diameter, and CTR), and one laboratory indicator (CEA). Although various independent risk factors in this model have been previously reported (41–50), not one has yet included CTR along with clinical and laboratory indicators to predict the malignancy of SPNs measuring ≤ 2 cm.

Some patients have clinical features that are considered risk factors for lung malignancy, such as advancing age, sex, smoking history, and chronic obstructive pulmonary disease (24, 41, 42, 51–55). Age has been shown to independently influence the malignancy of SPNs, and the risk of lung cancer incidence increases significantly with age (41, 42, 51, 54). The results of the present study are consistent with the above findings. In addition, sex is a major risk factor for the development of lung cancer, with women being more likely to develop lung cancer (24, 42, 51). Smoking history and COPD are also risk factors and promote the development and progression of lung cancer (55). In the present study, SPN malignancy did not differ significantly by sex, smoking history, lung function, and history of comorbid diseases including COPD, but this does not mean that these clinical characteristics are not associated with malignant SPNs. In future studies, the epidemiological factors of SPNs can be further explored by expanding the sample size, enriching the potential risk factors, and conducting multicenter prospective studies.

Some patients have hematological indicators that are considered risk factors for lung malignancy, such as tumor markers (43, 56–58). In addition, in recent years, the direction of research has gradually shifted to inflammatory factors (59–61). Several articles have demonstrated that inflammatory factors are associated with lung cancer prognosis (62–65). However, few articles have demonstrated that inflammatory factors are associated with lung carcinogenesis. Therefore, in the present study, we included not only tumor markers but also other hematological correlates, various types of leukocytes, and several inflammatory indicators derived from them. Inflammatory cells are an important component of the tumor microenvironment, and the inflammatory response plays a critical role in cancer development and progression and may be associated with systemic inflammation (19). Unfortunately, the present study did not investigate a definite association between inflammatory indicators and malignancy. Until now, studies have reported the association of inflammatory indicators with lung cancer prognosis and early recurrence (66–75). However, the association of inflammatory indicators with early lung carcinogenesis remains to be further investigated. However, no article has reported the association of inflammatory indicators with the development of early lung cancer. Inflammatory indicators may be normal in early-stage lung cancer. An association between the two could not be found in the present study. Among the various tumor markers, CEA is a polysaccharide protein complex involved in cell adhesion, which is usually absent or present in very small amounts in the blood of healthy adults and is thought to be associated with poor prognosis of tumors. Serum CEA levels are closely related to the pathological stage of lung cancer. Grunnet and Sorensen found that CEA was more significantly elevated in the serum of lung cancer patients than in patients with benign lesions (P< 0.05) (76). Our findings in which CEA was an independent predictor of malignant SPNs are consistent with previous findings (43, 44, 57, 76).

A number of additional imaging features also contribute to the risk stratification of patients with SPNs measuring ≤ 2 cm, including location, shape, spiculation, cavitation sign, calcification, vascular penetration sign, pleural adhesions, bronchus sign, lobulation, lymph node enlargement sign, pleural effusion sign, maximum tumor diameter, and CTR (77). We collected the above-mentioned imaging features of the patients, and after analysis, four independent predictors associated with the malignancy of SPNs measuring ≤ 2 cm were screened. Irregular nodules are a common finding in lung cancer screening (78, 79). Malignant nodules are more likely to have irregular, lobulated, or needle-like margins because of the spread of malignant cells within the lung mesenchyme and fibrosis within the tumor. Benign nodules are associated with smooth, rounded borders and exhibit a benign growth pattern. Calcification is a common CT feature of pulmonary tuberculosis and is usually considered a benign sign (79). Lung calcification results from deposition of calcium, mostly as a result of healing inflammation. Malignant tumors rarely have calcified foci, but mainly the nodules keep growing and clinically invade other healthy tissues. She et al. indicated that the risk of malignancy in SPNs increased 1.1-fold with a 1-mm increase in nodule diameter (80). However, Chen et al. did not find a diameter-related association with malignancy in small SPNs (81). Our study showed that the risk of malignancy positively correlated with the maximum diameter of the SPNs measuring ≤ 2 cm. CTR is currently the most commonly used indicator for the management of ground glass nodules (82). However, it is important to note that CTR is only an indicator for malignant nodules and it is generally used to predict the aggressiveness of nodules. It is generally accepted that a lower CTR corresponds to less aggressive behavior, while a higher CTR indicates a more aggressive tumor (83–88). A prospective radiological study for non-invasive prediction of pathological findings of clinical stage IA peripheral lung cancer by HRCT scan was conducted by the Japanese Clinical Oncology Study Group (JCOG0201) (27). The results of this study showed that pathological non-invasive carcinomas could be predicted by CTR values with a maximum tumor diameter ≤ 2 cm and a CTR ≤ 0.25, with a specificity of 98.7% for lung cancer. The 7.1-year follow-up results of this study concluded that both tumor maximum diameter ≤ 2 cm and CTR ≤ 0.25, and tumor maximum diameter ≤3 cm and CTR ≤ 0.5 on HRCT scans were good predictors of non-invasive pathology, with a 5-year overall survival rate of approximately 97% in both groups (89). In the present study, the role of CTR was contrary to previous findings, which may be because of the high number and proportion of *in situ* carcinomas with purely ground glass traits in the collected data.

The Mayo model was the most widely used model for predicting malignant SPN, and the PKUPH model claimed to be superior to traditional models. The Brock model is a more accurate predictive tool based on CT and clinical information description. However, these models did not involve clinical biomarkers. Foreign prediction models are not suitable for mainland Chinese populations. Some predictive models incorporate more advanced and quantitative imaging findings, such as CT attenuation and tumor diameter growth rates, in their assessments[5, 6]. However, these imaging data are rarely recognized and used by doctors since they are difficult to get, hard to conduct, and difficult to standardize. Our predictive model has the following advantages over previously published predictive models. First, we collected a relatively large number of small SPN cases and randomly divided them into a training cohort and an internal test cohort, which makes our conclusions more convincing. Second, surpassing previous work, we collected the most comprehensive clinical data and imaging data and provided a clear pathological diagnosis for each patient. Third, all important risk factors in the nomogram are available and prevalent in clinical practice. Fourth, the ROC, calibration, and DCA curves of the training cohort of the model perform well, and the accuracy and reliability of the model are satisfactory. Thus, our model can aid clinicians and facilitate a more individualized risk prediction for each patient.

There are some limitations to this study that need to be considered. First, owing to the retrospective nature of the study, we could not avoid potential selection bias. For example, we only included patients who underwent surgical resection in our department; otherwise, they would have been excluded, which is a selection bias. Second, our data were obtained from a single center with a relatively small sample size. The predictive model was only validated internally, so the selection bias present in the training cohort may also be present in the validation cohort. These reasons may limit the generalizability of our predictive nomogram and may also present some uncontrolled confounding factors. Therefore, the model requires further studies involving multiple centers and adequate samples to validate our results. Despite these limitations, the results of the internal validation suggest that the model will yield good results when applied to other populations. The independent risk factors identified in this study that preoperatively predict the probability of malignancy of SPNs measuring ≤ 2 cm, and the developed predictive nomogram may inform clinical decision-making by thoracic surgeons and pave the way for future research in this area.

## Conclusion

We developed a clinical nomogram for predicting the probability of malignancy of SPNs measuring ≤ 2 cm based on clinical and radiological characteristics, and the nomogram had good predictive performance. The nomogram could predict the probability of nodal malignancy in preoperative patients with SPNs measuring ≤ 2 cm, improving the diagnostic efficacy of lung malignancies and providing additional clinical reference information and diagnostic evidence to guide clinicians in the next step of intervention and subsequent treatment modalities.

## Data availability statement

The original contributions presented in the study are included in the article/**Supplementary Material**. Further inquiries can be directed to the corresponding authors.

## Ethics statement

The studies involving humans were approved by Institutional Review Board of Qilu Hospital of Shandong University (registration number: KYLL-202008-023-1). The studies were conducted in accordance with the local legislation and institutional requirements. The participants provided their written informed consent to participate in this study.

## Author contributions

Conceptualization: HT and MX. Methodology: RL. Software: MX. Validation: ZM and RL. Formal analysis: MX. Investigation: ZL and JL. Resources: ZL and JL. Data curation: WL and HZ. Writing—original draft preparation: MX. Writing—review and editing: MX and RL. Visualization: MX and HT. Supervision: HT and YT. Project administration: HT and YT. All authors contributed to the article and approved the submitted version.
